# Role of R-Loop Structure in Efficacy of RNA Elongation Synthesis by RNA Polymerase from *Escherichia coli*

**DOI:** 10.3390/ijms252212190

**Published:** 2024-11-14

**Authors:** Nadezhda A. Timofeyeva, Ekaterina I. Tsoi, Darya S. Novopashina, Aleksandra A. Kuznetsova, Nikita A. Kuznetsov

**Affiliations:** 1 Institute of Chemical Biology and Fundamental Medicine, Siberian Branch of Russian Academy of Sciences, Novosibirsk 630090, Russia; e.tsoi1@g.nsu.ru (E.I.T.); danov@niboch.nsc.ru (D.S.N.); sandra-k@niboch.nsc.ru (A.A.K.); 2 Department of Natural Sciences, Novosibirsk State University, Novosibirsk 630090, Russia

**Keywords:** RNA polymerase, R-loop, transcriptional pausing, enzymatic activity, processivity, enzyme kinetics

## Abstract

The mechanism of transcription proceeds through the formation of R-loop structures containing a DNA–RNA heteroduplex and a single-stranded DNA segment that should be placed inside the elongation complex; therefore, these nucleic acid segments are limited in length. The attachment of each nucleotide to the 3′ end of an RNA strand requires a repeating cycle of incoming nucleoside triphosphate binding, catalysis, and enzyme translocation. Within these steps of transcription elongation, RNA polymerase sequentially goes through several states and is post-translocated, catalytic, and pre-translocated. Moreover, the backward movement of the polymerase, which is essential for transcription pausing and proofreading activity, gives rise to a backtracked state. In the present study, to analyze both the efficacy of transcription elongation complex (TEC) formation and the rate of RNA synthesis, we used a set of model R-loops that mimic the pre-translocated state, post-translocated state, backtracked state, and a misincorporation event. It was shown that TEC assembly proceeds as an equilibrium process, including the simultaneous formation of a catalytically competent TEC as well as a catalytically inactive conformation. Our data suggest that the inactive complex of RNA polymerase with an R-loop undergoes slow conformational changes, resulting in a catalytically competent TEC. It was revealed that the structural features of R-loops affect the ratio between active and inactive states of the TEC, the rate of conformational rearrangements required for the induced-fit transition from the inactive state to the catalytically competent TEC, and the rates of accumulation of both the total RNA products and long RNA products.

## 1. Introduction

**Figure 1:**
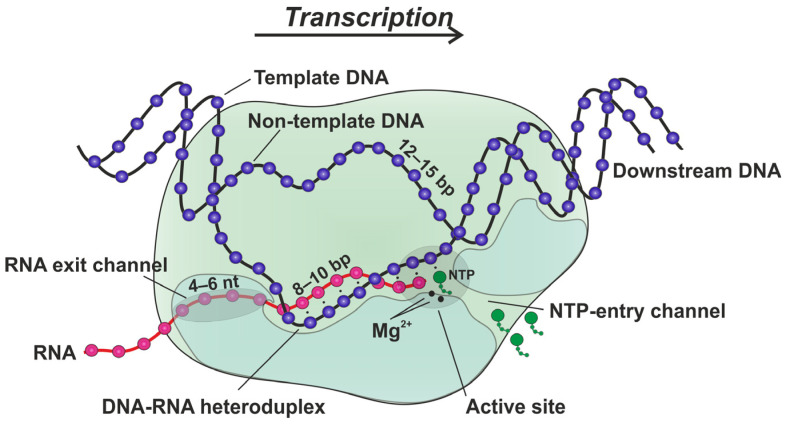
Structural features of the transcription elongation complex (TEC). The purple and magenta chains represent the DNA and RNA strands, respectively. RNA polymerase (RNAP) is depicted in light green. Nucleoside triphosphates (NTPs) are depicted in bright green. The key features of the TEC include (i) a downstream entry channel for DNA, (ii) a melted transcription bubble of 12–15 bp with an 8–10 bp DNA–RNA heteroduplex, (iii) an RNA exit channel that holds 4–6 nucleotides (nt) of single-stranded RNA, (iv) a secondary entry channel for NTP, and (v) the active site in which NTPs react with 3′-OH on RNA to extend the RNA strand.

The transcription process causes the formation of temporary transitional R-loop structures containing a DNA–RNA heteroduplex and a single-stranded DNA segment [1]. It is known that the length of the DNA–RNA hybrid inside the transcription elongation complex (TEC) typically does not exceed 10 bp, which is controlled by a special wedge-like structure of RNA polymerase (RNAP) [2,3]. The key features of the TEC (Figure 1) include (i) a downstream entry channel for DNA, (ii) a melted transcription bubble of 12–15 bp with an 8–10 bp DNA–RNA heteroduplex, (iii) an RNA exit channel that holds 4–6 nucleotides (nt) of single-stranded RNA, (iv) a secondary entry channel for nucleoside triphosphate (NTP), and (v) the active site in which NTPs react with 3′-OH on RNA to extend the RNA strand. The rehybridization of RNA with the template strand is blocked by the spatial separation of these strands from each other, leading to the formation of the DNA double-stranded structure that closes the R-loop after TEC translocation [1]. The TEC is typically stable, transcribing up to several thousand base pairs before release from the DNA template [4]. Nonetheless, RNAPs transcribe their templates at nonuniform velocities because the processive motion of the TEC is occasionally interrupted by a variety of pause states [5,6,7].

**Figure 2:**
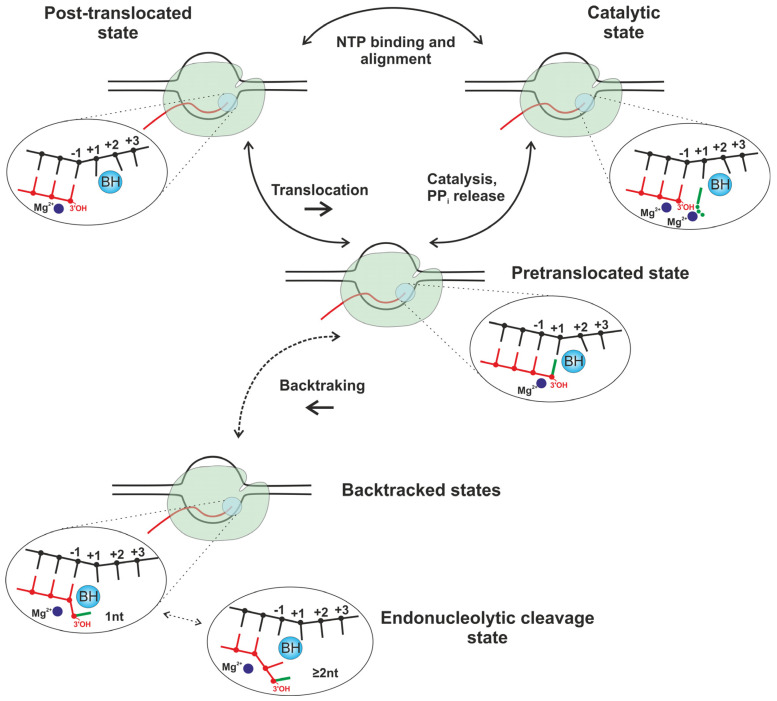
Key states of the transcription elongation mechanism. Black and red lines represent the DNA and RNA strands, respectively. RNAP is depicted in light green. NTP and nucleobase of newly incorporated nucleotide are depicted in bright green. The bridge helix of RNAP is schematically shown as BH. PP_i_ is pyrophosphate.

The mechanism of transcription elongation includes the repetition of a nucleotide addition cycle, which involves three essential steps (Figure 2): NTP entry and binding to the post-translocated state of the TEC, resulting in the catalytic state, nucleotide incorporation into the nascent transcript via phosphodiester bond formation with the displacement of pyrophosphate (PP_i_), leading to the pre-translocated state, and translocation of the nucleic acid scaffold in relation to the RNAP main frame to free up the NTP insertion site; this process is accompanied by transition to the post-translocated state, closing the cycle of transformations [1].

Indeed, the forward movement of polymerase (in the direction of transcription) by one nucleotide step along the DNA template from the post-translocated state makes the active site available for entry of the next NTP. The forward movement of the transcribing polymerase is driven by NTP binding, which captures the complex in the post-translocated state [8,9]. By contrast, the backward movement of polymerase (in the direction opposite to transcription) extrudes the last attached nucleotide from the active site, thereby forming backtracked states with one or more nucleotides (Figure 2). Transition to the 2-nt backtracked state allows the endonucleolytic cleavage of the transcript to proceed.

The elongating enzyme is thought to oscillate by simple diffusion between pre- and post-translocated states and occasionally arising backtracked states [4,10,11]. Structural data have revealed that between these states, important enzyme parts undergo conformational and interdomain movements. These parts are the trigger loop and the bridge helix (BH) (which are involved in translocation and 3′-end proofreading [3,12,13]), as well as the lid and the flap domains, which contact RNA and upstream DNA [14,15]. The emergence of multiple protein–nucleic acid contacts in the TEC and the ability of protein domains to sculpt the R-loop and spatially organize adjacent RNA and DNA strands raise an important question about how R-loop structures can affect the catalytic activities of the TEC. Therefore, in the present study, we used a set of model R-loops that mimic the pre-translocated state, post-translocated state, backtracked state, and a misincorporation event to analyze both the efficacy of TEC formation and the rate of RNA synthesis.

## 2. Results

### 2.1. The Rationale for Model R-Loops

In this study, we designed a set of R-loops that mimic nucleic acid structures in the pre-translocated, post-translocated, or backtracked state (Table 1). The pre-translocated state arises after the chemical attachment of the incoming NTP and can vary in the length of the R-loop bubble and the length of the RNA–DNA heteroduplex. In R-loop-9, the length of the RNA–DNA heteroduplex is 9 nt; therefore, it allows the natural transcription R-loop structure to form. Intermediate R-loops (R-loop-11 and R-loop-11Comp) contain an 11-nt RNA–DNA heteroduplex with noncomplimentary or complementary nucleotides between the template and nontemplate strands near the 3′ end of the RNA. These R-loops represent a transition state where the 11-nt length of the heteroduplex slightly exceeds the size of the natural heteroduplexes in the transcriptional R-loops (typically 8–10 nt). The subsequent increase in the length of the RNA–DNA heteroduplex in the R-loop up to 16, 21, or 31 nt gives rise to aberrant R-loop structures, which are not typical for transcription and cannot be placed within the RNAP internal channels controlling the size of the bubble and the RNA–DNA heteroduplex (Figure 1). Additionally, Loop-31Q was used, which forms a G4-quadruplex structure in the nontemplate strand. Furthermore, the post-translocated state of the TEC was modeled by R-loop-12, which contains an 11-nt heteroduplex and 12-nt bubble. In addition, two backtracked states were tested, where two or three 3′-terminal nucleotides of the RNA strand protruded out of the bubble structure (R-loop-11B2 and R-loop-11B3).

Downstream sequences of the template DNA strands were the same in all R-loops containing fully complementary RNA–DNA heteroduplexes. Upstream sequences of the template DNA strands were also the same in the vicinity of the 3′-end of the RNA primer in these R-loops. Thus, we believe that the differences in the efficacy of TEC formation and the kinetic parameters of any mutual conformational changes within the TEC and RNA elongation may only be caused by the differences in the secondary structure of R-loops containing complementary RNA–DNA heteroduplexes. In the case of R-loops where two or three 3′-terminal nucleotides of the RNA strand protruded out of the bubble structure, the TEC’s formation efficacy and the kinetic parameters may be affected by the backtracked state of the TEC and the noncomplementary 3′-terminal ribonucleotides, which should be cut prior to RNA elongation.

### 2.2. Formation of the Pre-Translocated Complex of RNAP with an R-Loop

**Figure 3:**
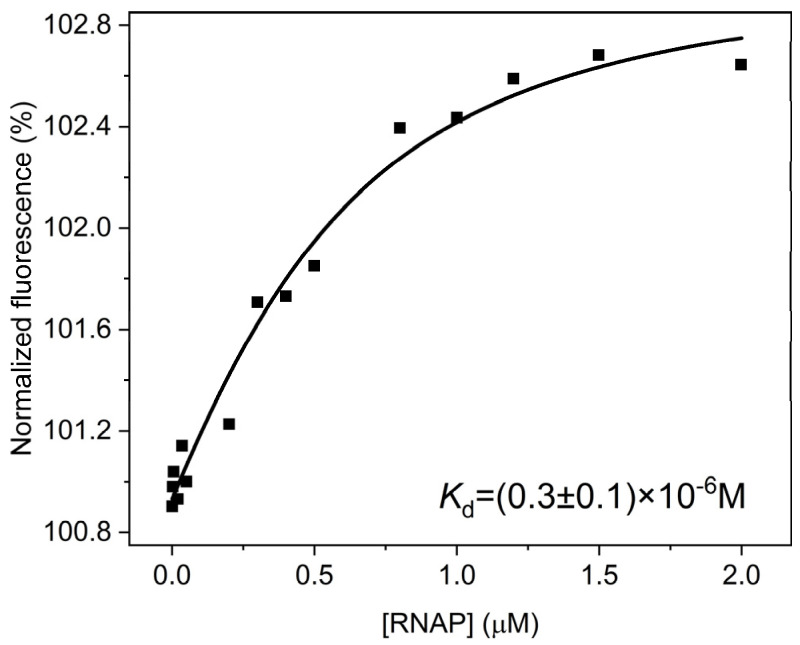
The titration curve characterizing the efficiency of RNAP binding to R-loop-9. Solid squares depict the plot of the normalized fluorescence signal (obtained in the microscale thermophoresis (MST) assay) of the TEC consisting of RNAP and R-loop-9 (0.5 μM) vs. RNAP concentration. Data were fitted to Equation (1) and yielded a dissociation constant (*K*_d_) of 0.3 ± 0.1 μM. Smooth curve is a result of the fitting procedure.

First of all, the efficiency of TEC assembly with the model R-loops was analyzed without NTP to prevent an RNA extension reaction. It was found that all tested R-loops, except R-loop-9, underwent 3′-terminal endonucleolytic degradation of the RNA strand in the presence of magnesium ions. Therefore, the endonucleolytic degradation of the RNA strand did not allow us to directly determine the dissociation constant of the TEC consisting of RNAP and most R-loops. The complex of RNAP with R-loop-9 is the only TEC whose dissociation constant could be determined directly because this TEC did not show endonucleolytic degradation of the RNA strand. To determine the efficiency of RNAP binding to R-loop-9, which contains a 9-nt bubble, we performed a microscale thermophoresis (MST) assay (Figure 3). The data indicate that under our experimental conditions, the TEC formation proceeds as an equilibrium process. The data were fitted to Equation (1) (see Section 4.3) and yielded a dissociation constant (*K*_d_) of 0.3 ± 0.1 μM. This dissociation constant characterizes the overall RNAP complex with R-loop-9, irrespective of whether it is catalytically competent.

**Figure 4:**
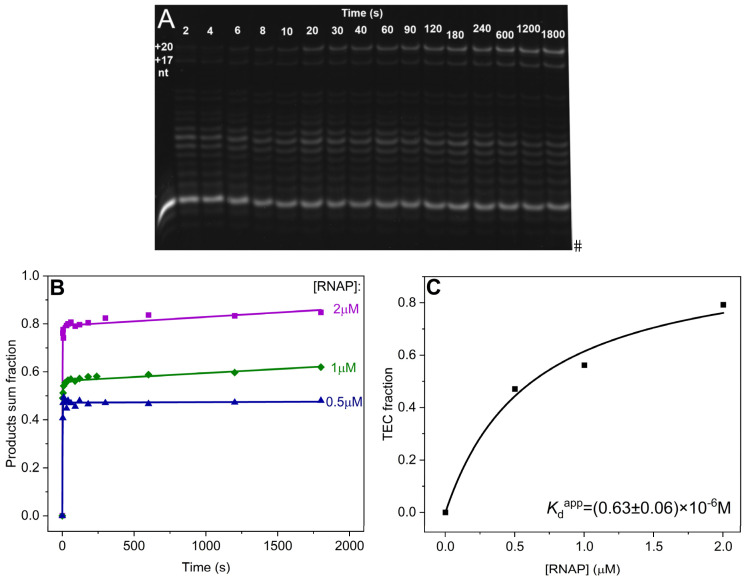
The estimation of the dissociation constant characterizing the catalytically competent TEC containing R-loop-9. (**A**) Polyacrylamide gel electrophoresis (PAGE) analysis of the time course of RNA extension in R-loop-9 (0.5 μM) by RNAP (1 μM). Time intervals for the enzymatic reaction are indicated above the corresponding rows. Bands corresponding to the +17 and +20 nt products are indicated on the left. (**B**) The time courses of RNA extension by 1–20 nt in R-loop-9 (0.5 μM). Concentrations of the enzyme are indicated next to the right axis. Smooth curves are the result of the fitting procedure according to Equation (2). The amplitude of an initial burst phase corresponds to the proportion of an initial catalytically competent TEC. (**C**) Dependence of the TEC proportion representing an initial burst of product accumulation on the RNAP concentration in the reaction mixture. Data were fitted to Equation (3) and yielded a dissociation constant (*K*_d_) of 0.63 ± 0.06 μM. Smooth curve is a result of the fitting procedure.

To estimate the dissociation constant characterizing the catalytically competent TEC, we analyzed the kinetics of RNA extension in R-loop-9 in the presence of NTPs and various concentrations of RNAP (Figure 4A). It turned out that the kinetics of RNA elongation have burst-like traces (Figure 4B). The fast accumulation of the RNA product for periods <60 s corresponded to the burst phase. The amplitude of the burst phase corresponds to the concentration of the catalytically competent TEC under the reaction conditions. The burst phase was followed by a slow accumulation of the RNA product. The time courses of RNA extension in R-loop-9 by RNAP (Figure 4B) were fitted to Equation (2) (see Section 4.5). The data clearly show that the amplitude of the burst phase, which is the measure of the concentration of the catalytically competent TEC in the reaction, is dependent on the concentration of RNAP. The dependence of the proportion of the catalytically competent TEC on RNAP concentration had a hyperbolic shape and was fitted to Equation (3) (see Section 4.5) to obtain an apparent dissociation constant (*K*_d_^app^) of 0.63 ± 0.0.06 μM, characterizing the stability of the catalytically competent TEC, involving RNAP and R-loop-9 (Figure 4C).

The apparent dissociation constant obtained for the catalytically competent TEC of RNAP with R-loop-9 by this approach was two-fold higher than the dissociation constant of the overall RNAP complex with this R-loop obtained by direct measurements in the MST assay. These results imply that the concentration of the catalytically competent TEC is lower than that of the overall RNAP complex with the R-loop at the initial moment.

We believe that some proportion of RNAP complexes with an R-loop initially has active conformation, resulting in the catalytically competent TEC. The remaining proportion of the enzyme–substrate complex in the mixture initially has catalytically inactive conformation. Thus, the burst phase of the product accumulation in the kinetic curves (Figure 4B) matches RNA synthesis in a processive mode within the catalytically competent TEC. The slow accumulation of the product after the burst phase can be explained by mutual conformational changes (induced fit) within the inactive complex of RNAP with an R-loop, leading to the formation of the catalytically competent TEC. This slow induced fit could be affected by the structural features of an R-loop, including the effects of the size of the bubble, the length of the RNA/DNA heteroduplex, and the G4-quadruplex in nontemplate strand.

### 2.3. The Effect of the R-Loop Structure on the Rate of RNA Extension by the TEC

**Figure 5:**
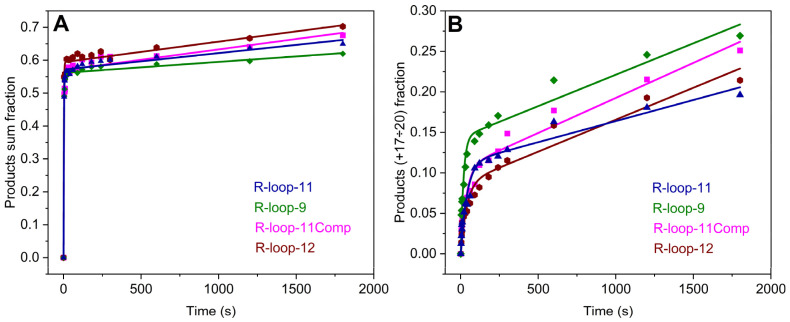
The time courses of RNA extension by 1–20 nt (**A**) and 17–20 nt (**B**) in model R-loops (0.5 μM) containing bubbles of 9–12 nt in length. Time courses corresponding to different R-loops are depicted in different colors. Concentration of the enzyme was 1 μM. Time courses were fitted to Equation (2). Smooth curves are the results of the fitting procedure.

To compare TEC formation efficiency and the rate of RNA extension by RNAP in the pre-translocated structures R-loop-9, R-loop-11, and R-loop11Comp and the post-translocated structure R-loop-12, kinetic traces of the accumulation of total products from +1 to +20 nt were obtained (Figure 5A). The time courses were fitted to Equation (2). It can be concluded that differences in structure among these R-loops do not affect the concentration of the catalytically competent TEC (corresponding to TEC formation efficiency) and the rate of the burst phase (corresponding to RNA synthesis in a processive mode within the catalytically competent TEC) since these parameters were very similar among all R-loop types (Table 2). It should be noted that the observed rate constant of the burst phase is not the rate constant of an individual reaction step. This rate constant characterizes the process of RNA elongation, which includes a repetition of a nucleotide addition cycle, translocation, pausing, and backtracking of RNAP. The observed rate of the slow linear phase was found to increase two-fold from R-loop-9 to R-loop-12, suggesting that the initial structure of R-loops affects the induced fit (leading to the formation of the catalytically competent TEC) within the inactive complex of RNAP with an R-loop.

Analysis of the kinetics of the incorporation of the last four nucleotides in the template (products from +17 to +20 nt) enabled us to compare the efficiency and rate of elongation near the end of the RNA extension. At this time point, the effect of the initial R-loop structure should be the most noticeable due to the multiple repetitions of the nucleotide addition cycle, which allowed the accumulation of variations in the efficiency at each nucleotide addition step (Figure 5B). The time courses of the accumulation of products from +17 to +20 nt were also fitted to Equation (2). The amplitude of the burst phase represented the TEC proportion, where the RNA primer was elongated up to the edge of the DNA template; this parameter corresponded to the efficiency of elongation up to the edge. This amplitude was the largest for the natural structure (R-loop-9) but slightly lower for the “transient size” in R-loop-11, R-loop11Comp, and R-loop-12. Moreover, the burst rate constant was approximately two-fold higher for naturally sized R-loop-9 (Table 2), supporting that the structural features of an R-loop within the catalytically competent TEC affect the pre-steady-state accumulation of long RNA products. On the other hand, the observed rate constants of the slow phase were very similar among all R-loop types, indicating that the rates of the processes within this phase corresponding to the steady-state accumulation of long RNA products are similar for these R-loops during RNA synthesis reaching the edge of the template strand. The induced fit within the inactive complex of RNAP with an R-loop has little effect on the slow phase of the steady-state accumulation of products from +17 to +20 nt since the observed rate constants of this phase are higher than that of the slow phase of total product accumulation (Table 2). Thus, the slow steady-state accumulation of products from +17 to +20 nt in R-loops can mainly be affected by RNAP backtracking and pausing within the catalytically competent TEC and the effect of the edge of the template strand.

### 2.4. The Effect of Backtracked R-Loop Structures on the Rate of RNA Extension by the TEC

**Figure 6:**
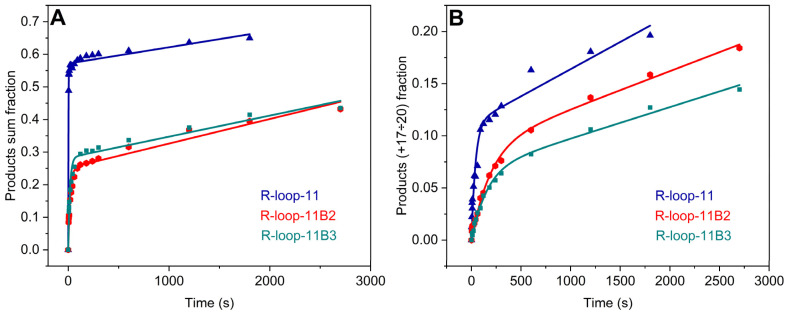
The time courses of RNA extension by 1–20 nt (**A**) and 17–20 nt (**B**) in R-loops (0.5 μM) containing a 3′-noncomplementary edge in an RNA primer. Time courses corresponding to different R-loops are depicted in different colors. Concentration of the enzyme was 1 μM. The time course of RNA extension in R-loop-11 is presented as a reference. Time courses were fitted to Equation (2). Smooth curves are the results of the fitting procedure.

Two types of backtracked R-loop structures containing two or three noncomplementary 3′-terminal nucleotides of the RNA strand protruding out of the bubble structure were employed to estimate their effects on TEC formation efficiency and the rate of RNA extension. As shown in Figure 6A, RNA synthesis in backtracked R-loops also proceeded in burst-like kinetics, however, with a 2-fold decrease in the efficiency of catalytically competent TEC formation and an 8–9-fold decrease in the observed rate of the burst phase of product accumulation when the total products (from +1 to +20 nt) were analyzed (Table 3). The decrease in the rate constant of the burst phase is caused not only by the two-fold decrease in the concentration of the catalytically competent TEC but also by the structural features of the backtracked R-loops since this constant decreased disproportionately to the decrease in the concentration of the catalytically competent TEC. The rate constants of RNA synthesis within the slow linear phase of product accumulation were found to increase 1.5- and 1.3-fold in the case of the backtracked states of the initial structures if compared to R-loop-11 and both backtracked types R-loop-11B2 and R-loop-11B3. This result supports again that the initial structure of R-loops affects the induced fit within the inactive complex of RNAP, with an R-loop leading to the formation of the catalytically competent TEC.

Analysis of the kinetics of the incorporation of the last four nucleotides in the template also reveals a slight decrease in the TEC proportion, where the RNA primer was elongated up to the edge of the DNA template for R-loop-11B2 and R-loop-11B3 (Figure 6B, Table 3). The rate constant of the burst phase was found to decrease up to 5-fold. The decrease in this rate constant is caused not only by a two-fold decrease in the concentration of the catalytically competent TEC but also by the strong effect of the backtracked structures on the rate of pre-steady-state accumulation of the long RNA products. The rate constant of the slow phase of 17–20 nt product accumulation was found to decrease 1.4- and 1.7-fold for backtracked R-loop-11B2 and R-loop-11B3, respectively. This slow phase was mainly affected by the processes that took place within the catalytically competent TEC. The decrease in the rate constant of this phase is caused by a two-fold decrease in the concentration of the catalytically competent TEC. Indeed, the total concentration of the substrates was the same, while the concentration of enzymes within the catalytically competent complexes with backtracked R-loops decreased two-fold, and the observed rate of the slow accumulation of the products decreased slightly less than two-fold under these conditions. Consequently, the absolute rates of the slow steady-state enzymatic synthesis of long products were comparable for R-loop-11 and backtracked R-loops.

### 2.5. The Effect of Bubble Size and Length of RNA/DNA Heteroduplex on the Rate of RNA Extension by the TEC

**Figure 7:**
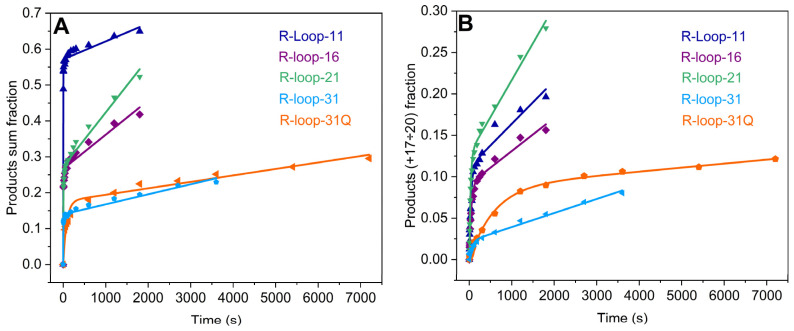
The time courses of RNA extension by 1–20 nt (**A**) and 17–20 nt (**B**) in R-loops (0.5 μM) containing a long bubble of 16, 21, or 31 nt in length. Time courses corresponding to different R-loops are depicted in different colors. Concentration of the enzyme was 1 μM. The time course of RNA extension in R-loop-11 is presented as a reference. Time courses were fitted to Equation (2). Smooth curves are the results of the fitting procedure.

To elucidate the role of the bubble size, a set of R-loops containing a long bubble of 16, 21, or 31 nt in length was used (Figure 7). In comparison with R-loop-11 containing an 11 nt bubble, the increase in bubble length led to a decrease in the efficiency of catalytically competent TEC formation (Table 4). Moreover, the active TEC proportion depended on the length of the bubble, decreasing approximately two-fold in the cases of 16 and 21 nt bubbles and four-fold for R-loop-31. In addition, the observed rate constants of the burst phase decreased slightly more than two-fold for all R-loops containing long bubbles. The decrease in the observed rate constant of this phase is probably affected by the decrease in the concentration of the catalytically competent TEC. On the other hand, the rate of the slow phase smoothly increased in the range of 11 nt → 16 nt → 21 nt but significantly dropped for 31 nt. These results suggest that the induced fit within the inactive complex of RNAP with an R-loop, leading to the formation of the catalytically competent TEC, is stimulated by long bubble structures up to a certain length, which probably can be properly placed in the RNAP channel.

Analysis of the kinetics of the incorporation of the last four nucleotides in the template (Figure 7B) reveals the most significant decrease in the proportion of TEC where the RNA primer was elongated up to the edge of the DNA template, as well as of the observed rates of the burst and linear phases for R-loop-31 containing the 31 nt bubble. In the cases of shorter bubbles (11, 16, and 21 nt), these parameters were close. Taking into account the last fact, while the concentration of the catalytically competent TEC decreased two-fold in the cases of R-loops containing 16 and 21 nt bubbles, we can conclude that the absolute elongation rates increase in such long bubble structures. The concentration of the catalytically competent TEC decreases four-fold, but the rates of the burst and linear phases decrease only 1.7 and 3-fold, respectively, in the case of R-loop-31. Thus, we can propose that the absolute elongation rates also increase in the long bubble structure containing 31 nt. In comparison with R-loop-31, the formation of the G4-quadruplex in the nontemplate strand did not significantly change the catalytically competent TEC proportion, but the observed rate of the burst phase of product accumulation decreased 17-fold when the total products were analyzed. The rate constant of the slow phase decreased 1.5-fold. Analysis of the last four nucleotide incorporation reveals a 9-fold decrease in the rate constant of the burst phase and a 3.5-fold decrease in the rate constant of the linear phase. These results indicate the strong effect of the quadruplex not only on the rate of total product accumulation but also on the rate of accumulation of the long RNA products (Figure 7, Table 4). Thus, our data reveal that the complicated structure in the nontemplate strand does not noticeably affect the TEC formation efficiency but slightly decreases the rate of induced fit within the inactive complex and significantly slows down the RNA elongation, probably preventing the proper positioning of R-loops in the RNAP channel.

## 3. Discussion

In summary, this work enables us to outline the effect of R-loop structures on TEC formation efficiency and the rate of RNA extension by *E. coli* RNAP. As follows from the data described above, the TEC assembly proceeds as an equilibrium process. *E. coli* RNAP generates the initial complex with the R-loop, some proportion of which initially has active conformation, resulting in the catalytically competent TEC. The remaining proportion of the enzyme–substrate complex initially has catalytically inactive conformation. In accordance with the concept of induced fit, the inactive complex of RNAP with the R-loop undergoes mutual conformational changes, leading to a thermodynamically favorable catalytically competent TEC [16,17].

The data obtained in this work indicate that the induced fit within the inactive complex of RNAP is affected by the structural features of R-loops. The observed rate of this process is found to increase two-fold from pre-translocated R-loop-9 to post-translocated R-loop-12 and is found to increase 1.5- and 1.3-fold in the cases of the backtracked states of the initial structures when compared to R-loop-11 and both backtracked types R-loop-11B2 and R-loop-11B3 (containing two or three noncomplementary 3′-terminal nucleotides). The induced fit is stimulated by a long bubble structure up to a certain length (at least up to 21 nt), which, probably can be properly placed in the RNAP channel. On the other hand, the rate of this process significantly drops for the 31 nt bubble structure. The G4-quadruplex in the nontemplate strand of the R-loop with a 31-nt bubble structure additionally decreases the rate of induced fit within the inactive complex.

Our data reveal that the concentration of the catalytically competent TEC (corresponding to TEC formation efficiency) is not affected by minor differences in the structures of pre-translocated R-loop-9, R-loop-11, and R-loop11Comp and post-translocated R-loop-12. At the same time, our data show that the formation efficiency of the catalytically competent TEC decreases two-fold in the case of backtracked R-loop structures containing two or three noncomplementary 3′-terminal nucleotides of the RNA strand protruding out of the bubble structure. The catalytically competent TEC proportion also depends on the length of the bubble within the R-loop, decreasing approximately two-fold in the cases of 16 and 21 nt bubbles and four-fold in R-loop-31.

The rate constant of the burst phase in the kinetic traces of the accumulation of total products characterizes the pre-steady-state process of RNA elongation within the catalytically competent TEC. The elongation includes a repetition of a nucleotide addition cycle, translocation, pausing, and backtracking of RNAP. The rate of the burst phase is not affected by minor differences in the structures of pre-translocated R-loop-9, R-loop-11, R-loop11Comp and post-translocated R-loop-12. In addition, we believe that the absolute rate of the polymerase reaction within the catalytically competent TEC does not depend on the length of the bubble within the R-loop since the decrease in the corresponding observed rate constants of this phase is probably caused by the decrease in the concentration of the catalytically competent TEC. On the other hand, this rate 8–9-fold decreases in the cases of backtracked R-loop structures containing two or three noncomplementary 3′-terminal nucleotides of the RNA strand. The latter decrease is caused not only by the decrease in the concentration of the catalytically competent TEC but also by the structural features of the backtracked R-loops. In addition, complicated structures, such as G4-quadruplex, in the nontemplate strand significantly influence the rate of the polymerase reaction, reducing it 17-fold. Analysis of the kinetics of the incorporation of the last four nucleotides in the template enables us to compare the efficiency and rates of RNA elongation up to the edge of the DNA template. The TEC proportion (corresponding to efficiency of elongation up to the edge of the DNA template) where the RNA primer is elongated up to the edge of the DNA template is the largest for the natural structure (R-loop-9) but slightly lower for the “transient size” in R-loop-11, R-loop11Comp, R-loop-12, and backtracked R-loop structures containing two or three noncomplementary 3′-terminal nucleotides of the RNA strand. We observed the most significant decrease in this proportion of TEC for R-loop-31, whereas the increase in the bubble size from 11 to 21 nt does not lead to significant changes.

Unexpectedly, our data reveal that the structural features of R-loops within the catalytically competent TEC affect the pre-steady-state accumulation of long RNA products. Indeed, the rate constant of this accumulation is approximately two-fold higher for naturally sized R-loop-9 in comparison with the “transient size” in R-loop-11, R-loop11Comp, and R-loop-12. This rate constant is reduced up to 5-fold in the case of backtracked R-loop structures containing two or three noncomplementary 3′-terminal nucleotides of the RNA strand. The rate decrease in the latter case is caused not only by the decrease in concentration of the catalytically competent TEC but also by the strong effect of the backtracked structures on the rate of the pre-steady-state accumulation of long RNA products. In addition, the formation of the G4-quadruplex in the nontemplate strand of R-loop-31 containing the 31 nt bubble structure reduces this rate constant 9-fold.

On the other hand, the observed rate constants of the steady-state accumulation of long RNA products were very similar among pre-translocated R-loop-9, R-loop-11, R-loop11Comp, and post-translocated R-loop-12. In addition, we propose that the absolute rates of the slow steady-state enzymatic synthesis of long products were comparable for R-loop-11 and backtracked R-loops containing two or three noncomplementary 3′-terminal nucleotides of the RNA strand. Moreover, we believe that the absolute elongation rates of the steady-state, as well as the pre-steady-state, accumulation of long RNA products increase in long bubble structures that are at least up to 31 nt in bubble length. The formation of the G4-quadruplex in the nontemplate strand of R-loop-31 slowed steady-state accumulation 3.5-fold.

## 4. Materials and Methods

### 4.1. Oligonucleotides Synthesis

The synthesis of the oligodeoxyribonucleotides and oligoribonucleotides (Table 1) was carried out on an ASM-800DNA/RNA synthesizer (Biosset, Novosibirsk, Russia). The oligodeoxyribonucleotides were purchased from Biosset. FAM-labeled oligoribonucleotides were synthesized by means of standard commercial phosphoramidites and CPG solid supports from Glen Research (Sterling, VA, USA) in the Laboratory of RNA Chemistry (ICBFM SB RAS, Novosibirsk, Russia). The oligonucleotides were deprotected according to the manufacturer’s protocols and were purified by high-performance liquid chromatography or denaturing 20% polyacrylamide gel electrophoresis (PAGE). The homogeneity of all used oligonucleotides was checked by PAGE. The concentrations of oligonucleotides were calculated from their absorbance at 260 nm (A_260_).

### 4.2. Enzyme Purification

*Escherichia coli* core RNAP was expressed and purified from BL21(DE3) cells transformed by a pVS10-based plasmid encoding all core RNAP subunits, as described previously [18,19].

Briefly, RNAP was isolated from *E. coli* BL21(DE3) cells transformed with plasmid pVS10 carrying a relevant N-terminal His-tagged gene construct. To purify the enzyme expressed as recombinant proteins, 2 L of culture (in LB broth) of *E. coli* cells carrying the encoding vector construct was grown with 100 µg/mL ampicillin at 37 °C until A_600_ reached 0.5–0.7; the expression of the enzyme was induced during 3 h at 37 °C with 1 mM isopropyl β-D-1-thiogalactopyranoside. The cells were harvested by centrifugation (4000 RPM, 20 min at 4 °C) in a Drawell GL-22MC centrifuge (Drawell, Chongqing, China), and the cell pellets were stored at −20 °C. Before proceeding with protein purification, the cell pellets were thawed on ice and resuspended in 30 mL of cell lysis buffer, 50 mM Tris-HCl (pH 7.9), 233 mM NaCl, 2 mM EDTA, 5% glycerol, 0.2% Tween-20, 1 mM β-mercaptoethanol, and a protease inhibitor cocktail (Macklin, Shanghai, China), followed by cell lysis by means of a French press. All the purification procedures were carried out at 4 °C. The homogenate was centrifuged at 15,000 RPM for 15 min. Then, the supernatant was centrifuged under the same conditions. A 5% solution of Polymin P (pH 7.0) was added to the supernatant dropwise to a final concentration of 0.35% with permanent stirring. The obtained suspension was incubated on ice for 15 min and then centrifuged at 15,000 RPM for 15 min. The precipitate was resuspended in 10 mL of buffer 10 mM Tris-HCl (pH 7.9), 300 mM NaCl, 0.1 mM EDTA, 0.1 mM dithiothreitol, and 5% glycerol and then was centrifuged at 15,000 RPM for 15 min. The precipitate was resuspended in 12 mL of buffer 10 mM Tris-HCl (pH 7.9), 1 M NaCl, 0.1 mM EDTA, 0.1 mM dithiothreitol, and 5% glycerol and then was centrifuged under the same conditions. Ammonium sulfate was added to the supernatant to a final concentration of 350 g/L with permanent stirring. The obtained suspension was stirred at 4 °C for 40 min and then was stored on ice overnight. The suspension was centrifuged at 15,000 RPM for 15 min the next day. The precipitate was resuspended in 12 mL of buffer, 10 mM Tris-HCl (pH 7.9), 0.1 mM EDTA, 0.1 mM dithiothreitol, and 5% glycerol and then was centrifuged at 10,000 RPM for 15 min. The supernatant was filtered through a 0.45 um syringe filter (Labfil, Hangzhou, China). The supernatant was diluted twice with a buffer, 10 mM Tris-HCl (pH 7.9), 0.1 mM EDTA, 0.1 mM dithiothreitol, and 5% glycerol and loaded on a 5 mL HiTrap-Heparin^TM^ column (Cytiva GE Healthcare Life Sciences, Marlborough, MA, USA) pre-equilibrated in a buffer, 20 mM Tris-HCl (pH 7.9), and 5% glycerol at a rate of 1 mL/min. The column was washed with 50 mL of a buffer, 20 mM Tris-HCl (pH 7.9), 5% glycerol then with 25 mL of a buffer, 20 mM Tris-HCl (pH 7.9), 450 mM NaCl, and 5% glycerol. The bound proteins were eluted with 25 mL of a buffer, 20 mM Tris-HCl (pH 7.9), 600 mM NaCl, and 5% glycerol. The fraction containing the enzyme was diluted with a buffer, 20 mM Tris-HCl (pH 7.9), and 5% glycerol to a final NaCl concentration of 500 mM and loaded on a 5 mL HiTrap-Chelating^TM^ column (Cytiva GE Healthcare Life Sciences, Marlborough, MA, USA) pre-equilibrated in a buffer, 10 mM Tris-HCl (pH 7.9) and 500 mM NaCl at a rate of 0.5 mL/min. The column was washed with 5 mL of a buffer, 10 mM Tris-HCl (pH 7.9), and 500 mM NaCl and then with 25 mL of a buffer, 10 mM Tris-HCl (pH 7.9) and 500 mM NaCl, 20 mM imidazole. The bound enzyme was eluted with 25 mL of a buffer, 10 mM Tris-HCl (pH 7.9), 500 mM NaCl, and 200 mM imidazole. The fractions containing RNAP were pooled and dialyzed against 1 L of dialysis buffer, 30 mM Tris-HCl (pH 7.9), 50 mM NaCl, 0.5 mM EDTA, 0.1 mM DTT, and 5% glycerol overnight. The dialyzed sample was diluted twice with a buffer, 40 mM Tris-HCl (pH 7.9), 1 mM EDTA, 0.1 mM DTT, and 5% glycerol and loaded on a 1 mL Monomix Mab60-Q column (Sepax Technologies, Suzhou, China) pre-equilibrated in the same buffer at a rate of 0.3 mL/min. The column was washed with 5 mL of a buffer, 40 mM Tris-HCl (pH 7.9), 1 mM EDTA, 0.1 mM DTT, and 5% glycerol. The bound enzyme was eluted with a 0→600 mM gradient of NaCl. The fractions containing RNAP were pooled and concentrated using a 100,000 MWCO JetSpinTM centrifugal filter (Biofil, Guangzhou, China). Glycerol and DTT were added up to 50% and 1 mM, respectively. The homogeneity of the enzyme was verified by SDS-PAGE; the enzyme concentration was measured using the Bradford method.

### 4.3. Microscale Thermophoresis (MST)

The stability constant of the transcription elongation complex between the RNAP and R-loop containing the 9 nt bubble was determined by means of a Monolith NT.115 system (NanoTemper Technologies, Yokohama, Japan) using standard capillaries (MonolithTM NT.115 Series, NanoTemper Technologies, Yokohama, Japan).

Each point of the titration curve was determined by measuring the fluorescence intensities of individual solutions (10 μL) containing an R-loop (0.5 μM) and the enzyme (0.0005–2 μM) in the reaction buffer, 10 mM MgCl_2_, 40 mM Tris-HCl (pH 7.9), and 40 mM KCl. The transcription elongation complex TEC (0.5 μM) was assembled, as described in [20]. To anneal the DNA/RNA heteroduplex, the mixture was incubated for 3 min at 70 °C and allowed to cool slowly to room temperature. A FAM-labeled RNA primer (0.5 μM) annealed to the template DNA (0.55 μM) was incubated with core RNAP (0.0005–2 μM) for 10 min at 25 °C in a buffer, 40 mM Tris-HCl (pH 7.9) and 40 mM KCl. Then, the nontemplate DNA (2.5 μM) was added and incubated for 20 min at 25 °C. After, Mg^2+^ was added to a final concentration of 10 mM. Three individual curves were averaged for the reported titration curve.

To calculate the dissociation constant, the experimental data were processed in OriginPro 2021 software (OriginLab Corp., Northampton, MA, USA; Equation (1)). The ratio between the normalized fluorescence *F* and *K*_d_ can be expressed as:

(1) $$F=f_{\mathrm{TEC}}{\left[L\right]}_{0}+\left(f_{L}-f_{\mathrm{TEC}}\right)\times \left\{\frac{{\left[L\right]}_{0}-{\left[E\right]}_{0}-K_{d}}{2}+\sqrt{{\left(\frac{{\left[L\right]}_{0}-{\left[E\right]}_{0}-K_{d}}{2}\right)}^{2}+K_{d}\;{\left[L\right]}_{0}}\right\}$$

where *f*_L_ and *f*_TEC_ are the specific fluorescence intensities of the free R-loop and the transcription elongation complex TEC; and [L]_0_ and [E]_0_ are the total amounts of the R-loop and protein, respectively [21].

### 4.4. Time Courses of RNA Extension

Transcription elongation complexes (TECs) (1 μM) were assembled, as described in [20]. To anneal the DNA/RNA heteroduplex, the mixture was incubated for 3 min at 70 °C and allowed to cool slowly to room temperature. A FAM-labeled RNA primer (1 μM) annealed to the template DNA (1.1 μM) was incubated with core RNAP (2 μM) for 10 min at 25 °C in a buffer, 40 mM Tris-HCl (pH 7.9), and 40 mM KCl. Then, the nontemplate DNA (2 μM) was added and incubated for 20 min at 25 °C.

The reaction was initiated by the rapid mixing of 1 μM TEC (in a buffer, 40 mM Tris-HCl (pH 7.9), and 40 mM KCl) with an equal volume of 200 μM NTPs (in a buffer, 20 mM MgCl_2_, 40 mM Tris-HCl (pH 7.9), and 40 mM KCl) at 25 °C. The reaction was quenched at designated intervals by 0.5 M EDTA. EDTA was removed from the samples using Sephadex G-25 (GE Healthcare, Uppsala, Sweden) saturated with 8 M Urea. RNA products were separated in 20% denaturing PAGE, visualized using the VersaDoc Imaging System 4000 MP (Bio-Rad, Hercules, CA, USA), and quantified using Gel-Pro40 Analyzer software (Media Cybernetics, Rockville, MD, USA). Two or three individual traces were averaged for each reported curve. The final composition of each reaction mixture was as follows: 0.5 μM RNA primer, 0.55 μM template DNA, 1 μM nontemplate DNA, 1 μM core RNAP, 100 μM NTPs in a reaction buffer, 10 mM MgCl_2_, 40 mM Tris-HCl (pH 7.9), and 40 mM KCl.

### 4.5. PAGE Data Analysis

The time courses of the RNA extension obtained in the PAGE analysis were fitted to a single exponential equation, followed by a linear one by means of OriginPro 2021 software (Originlab Corp., Northampton, MA, USA; Equation (2)).

*Product proportion* = A × [1 − exp(−*k*_obs_^1^ × t)] + *k*_obs_^2^ × t (2)

where A is the amplitude of an initial burst phase corresponded to the amount of an initial transcription elongation complex TEC, *k*_obs_^i^ (s^−1^) denotes the observed rate constants, and t represents reaction time.

The dependence of initial catalytically competent TEC proportion on the concentration of RNAP was fitted to hyperbolic Equation (3) and yielded an apparent dissociation constant (*K*_d_^app^):

TEC = [RNAP]/((*K*_d_^app^) + [RNAP]) (3)

where (*K*_d_^app^) is the apparent equilibrium constant of the TEC formation (M).

## 5. Conclusions

TEC assembly proceeds as an equilibrium process. *E. coli* RNAP generates the initial complex with an R-loop, some proportion of which initially has active conformation, resulting in a catalytically competent TEC. The remaining proportion of the enzyme–substrate complex initially has catalytically inactive conformation. The inactive complex undergoes mutual conformational changes (induced fit), leading to the formation of the catalytically competent TEC.

Minor differences in initial structure among pre-translocated and post-translocated R-loops affect the induced fit within the inactive complex and unexpectedly affect the rate of the pre-steady-state accumulation of long RNA products.

Backtracked R-loop structures containing two or three noncomplementary 3′-terminal nucleotides of the RNA strand decrease the efficiency of the catalytically competent TEC formation, slightly accelerate the induced fit within the inactive complex, slow down the pre-steady-state processes of accumulation of total RNA products as well as long RNA products.

Long bubble structures within R-loops decrease the efficiency of catalytically competent TEC formation (an active TEC proportion depends on the length of the bubble). Such structures up to a certain length (at least up to 21 nt) accelerate the induced fit within the inactive complex, but this process slows down significantly for the 31 nt bubble structure. Long bubble structures of at least up to 31 nt in bubble length probably increase the absolute rates of the pre-steady-state, as well as the steady-state, accumulation of long RNA products.

The formation of the G4-quadruplex in the nontemplate strand within an R-loop slightly decreases the rate of induced fit within the inactive complex, significantly slows down the pre-steady-state accumulation of total RNA products, and the pre-steady-state, as well as the steady-state, processes of the accumulation of long RNA products.

## Figures and Tables

**Table 1:** Sequences of oligonucleotides forming R-loops *.

R-Loops Containing Bubbles of 9–12 nt in Length	Shorthand	Type of Complex
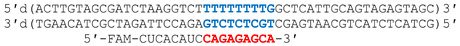	R-loop-9	Pre-translocated, 9-nt bubble
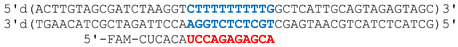	R-loop-11	Pre-translocated, 11-nt bubble
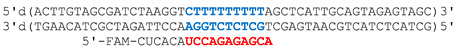	R-loop-11Comp	Pre-translocated, 11-nt bubble, 3′-terminal complementary base pair
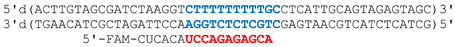	R-loop-12	Post-translocated, 12-nt bubble
R-loops containing a 3’ noncomplementary edge in an RNA primer		
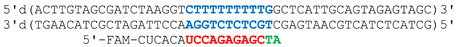	R-loop-11B2	2-nt backtracked, 11-nt bubble, 3′-terminal mismatch of 2 nt
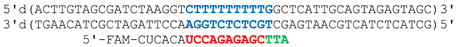	R-loop-11B3	3-nt backtracked, 11-nt bubble, 3′-terminal mismatch of 3 nt
R-loops containing a long bubble of 16, 21, or 31 nt in length		
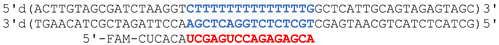	R-loop-16	Pre-translocated, 16-nt bubble
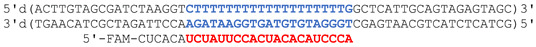	R-loop-21	Pre-translocated, 21-nt bubble
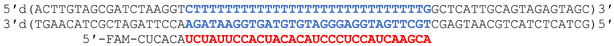	R-loop-31	Pre-translocated, 31-nt bubble
R-loop containing a quadruplex in nontemplate DNA		
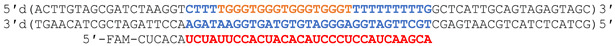	R-loop-31Q	Pre-translocated, 31-nt bubble, quadruplex

* The sequences forming a bubble are highlighted in blue, the RNA sequence forming a DNA–RNA heteroduplex is indicated by red, the mismatched sequence of the 3′ terminus of the RNA strand is highlighted in green, and the sequence forming a quadruplex is highlighted in orange.

**Table 2:** TEC formation efficiency and observed rate constants of RNA extension in R-loops containing bubbles of 9–12 nt in length.

R-Loops	RNA Extension by 1–20 nt			RNA Extension by 17–20 nt		
	*k*_1_^Sum^, s^−1^	*k*_2_^Sum^, s^−1^	[TEC]^Sum^, %	*k*_1_^17÷20^, s^−1^	*k*_2_^17÷20^, s^−1^	[TEC]^17÷20^, %
R-loop-9	0.48 ± 0.03	(3.3 ± 0.5) × 10^−5^	56.2 ± 0.4	0.057 ± 0.009	(7.8 ± 0.8) × 10^−5^	14.4 ± 0.7
R-loop-11	0.46 ± 0.03	(5.0 ± 0.6) × 10^−5^	57.2 ± 0.4	0.025 ± 0.004	5.2 ± 0.8) × 10^−5^	11.2 ± 0.7
R-loop-11Comp	0.42 ± 0.05	(6 ± 1) × 10^−5^	57.0 ± 0.8	0.028 ± 0.006	(8.7 ± 0.9) × 10^−5^	10.6 ± 0.8
R-loop-12	0.54 ± 0.07	(6 ± 1) × 10^−5^	59.4 ± 0.6	0.026 ± 0.006	(7.9 ± 0.8) × 10^−5^	8.7 ± 0.7

**Table 3:** TEC formation efficiency and observed rate constants of RNA extension in backtracked R-loops.

R-Loops	RNA Extension by 1–20 nt			RNA Extension by 17–20 nt		
	*k*_1_^Sum^, s^−1^	*k*_2_^Sum^, s^−1^	[TEC]^Sum^, %	*k*_1_^17÷20^, s^−1^	*k*_2_^17÷20^, s^−1^	[TEC]^17÷20^, %
R-loop-11	0.46 ± 0.03	(5.0 ± 0.6) × 10^−5^	57.2 ± 0.4	0.025 ± 0.004	(5.2 ± 0.8) × 10^−5^	11.2 ± 0.7
R-loop-11B2	0.051 ± 0.007	(7.5 ± 0.8) × 10^−5^	25.1 ± 0.9	(5.3 ± 0.7) × 10^−3^	(3.6 ± 0.4) × 10^−5^	8.9 ± 0.6
R-loop-11B3	0.059 ± 0.007	(6.5 ± 0.8) × 10^−5^	28 ± 1	(6.5 ± 0.6) × 10^−3^	(3.0 ± 0.2) × 10^−5^	6.7 ± 0.3

**Table 4:** TEC formation efficiency and observed rate constants of RNA extension in aberrant size R-loops.

R-Loops	RNA Extension by 1–20 nt			RNA Extension by 17–20 nt		
	*k*_1_^Sum^, s^−1^	*k*_2_^Sum^, s^−1^	[TEC]^Sum^, %	*k*_1_^17÷20^, s^−1^	*k*_2_^17÷20^, s^−1^	[TEC]^17÷20^, %
R-loop-11	0.46 ± 0.03	(5.0 ± 0.6) × 10^−5^	57.2 ± 0.4	0.025 ± 0.004	5.2 ± 0.8) × 10^−5^	11.2 ± 0.7
R-loop-16	0.18 ± 0.03	(9.5 ± 0.9) × 10^−5^	26.6 ± 0.6	0.023 ± 0.002	(4.0 ± 0.3) × 10^−5^	9.1 ± 0.3
R-loop-21	0.17 ± 0.03	(15.1± 0.9) × 10^−5^	27.3 ± 0.7	0.034 ± 0.003	(8.9± 0.4) × 10^−5^	12.8 ± 0.4
R-loop-31	0.20 ± 0.03	(2.8 ± 0.2) × 10^−5^	14.0 ± 0.3	0.015 ± 0.004	(1.7 ± 0.1) × 10^−5^	2.2 ± 0.2
R-loop-31Q	0.012 ± 0.002	(1.8 ± 0.3) × 10^−5^	18 ± 1	(1.7 ± 0.1) × 10^−3^	(0.48 ± 0.07) × 10^−5^	8.7 ± 0.3

## Data Availability

Data are available from N.A.T. (Tel. +7(383)363-5174, E-mail: na_timof@niboch.nsc.ru) and N.A.K. (Tel.: +7-(383)-363-5175, e-mail: nikita.kuznetsov@niboch.nsc.ru) upon request.
